# “‘It is very difficult in this business if you want to have a good conscience’: pharmaceutical governance and on-the-ground ethical labor in Ghana”: a letter to editors

**DOI:** 10.1080/11287462.2023.2268885

**Published:** 2023-10-18

**Authors:** Livia Maria de Souza Gonçalves, Felipe Felizardo Mattos Vieira, Ariadne Botto Fiorot, Sthefany Brito Salomão, Luciano Soares

**Affiliations:** a Federal University of Santa Catarina, Florianópolis, Brazil; b Federal University of Paraná, Curitiba, Brazil; c Federal University of Espírito Santo, Vitória, Brazil

**Keywords:** Access to medicines, essential medicines, universal health care, Africa, bioethical issues

## Abstract

Establishing effective pharmaceutical governance is a challenge for government agencies, private enterprises, and professionals working on the ground, demanding complex ethical decisions from the actors involved, especially in a lower-middle-income country like Ghana. This letter aims to share the author’s perspectives and additional considerations on the analyses of the reports in the paper “It is very difficult in this business if you want to have a good conscience”: *pharmaceutical governance and on-the-ground ethical labor in Ghana* by Hampshire et al. The letter's authors discuss the need to advance universal health coverage in Ghana, the everyday ethics, and the disparities between the collective and individual moral consciousness of the participants, as well as other aspects of governance in the pharmaceutical sector.

Dear Editor-in-Chief of the Global Bioethics Journal,

This letter aims to communicate the author’s perspectives and provide additional considerations on the topics covered in the paper published on pages 103–121 of volume 33, n° 1, 2022 of the journal in question, entitled “It is very difficult in this business if you want to have a good conscience”: *pharmaceutical governance and on-the-ground ethical labor in Ghana* by the authors: Kate Hampshire, Simon Mariwah, Daniel Amoako-Sakyi, and Heather Hamill (Hampshire et al., 2022).

The response in the present document represents the result of discussion and careful consideration of reports presented by Hampshire et al., by members of the higher education course “Ethics and Market in the Pharmaceutical Sector” of the Graduate Program in Pharmaceutical Services and Policies (PPGASFAR, in the Portuguese acronym), a graduate network program with Ph.D. and master's degree students involving several federal universities in Brazil.

Initially, we congratulate the authors for raising the discussion about such a relevant topic as “pharmaceutical governance” in low- and middle-income countries and bringing to light the decisions about ethical and moral conflicts that “front-line” professionals face when trying to guarantee the population access to medicines and healthcare products with quality and safety.

First, we have analyzed it from the perspective of “everyday ethics”, which according to Kingori is deeply rooted in social relations and depends on the “what”, “why” and “who” (Kingori, 2013). The influence of these points in the participants’ speechesis clear in the development of the article, reconstructing all judgments about what individuals should do and what they can do at any given moment.

In addition, we emphasize that, in our analysis, a crucial point for decision-making by governance agents reported in the article lies within the local social-political context, in which pharmacies and places that sell over-the-counter medicines are seen essentially as commercial institutions and health services are placed in the background. After all, health regulations and pharmacy councils in Ghana are centered on medicine as a raw product and not on its use.

In the light of Durkheim's paradigm (Mocelin & Azambuja, 2011), a comprehensive analysis of the social being of the participants described in the case reports allows to perceive that, despite the insertion of individuals in a capitalist society, in which the pharmaceutical market and health regulations encompass medicine as a raw product, there is a conflict between socially established values and individual interests. This conflict comprises a disputed space for principles – mainly between those related to commercial interests and the public health of the most vulnerable; i.e. the collective and individual conscience, which in the presented case is defined based on ethical reflection.

It is important to point out that the results of the study make clear that Ghana still has a long way to go before establishing an universal health coverage, a fundamental principle established in the Sustainable Development Goals (SDGs) for 2030 of the United Nations (United Nations, 2015). Our argument is based on our experience and research on the Brazilian Unified Health System (SUS, in the Portuguese acronym), an essential public policy in Brazil that has contributed to reducing the unparalleled social inequality and to the consolidation of democracy in the country (Castro et al., 2019; Massuda et al., 2023).

There is an urgent need for global health institutions and the state of Ghana to work together to developpe strategies to build effective public health policies to finance and structure the selection, programming, procurement, storage, distribution, and disposal of medicines. Such efforts must also include granting the offer of other pharmaceutical services, insuring access to quality essential healthcare services and safe, effective, quality and affordable access to essential medicines for all, especially in a lower-middle-income country like Ghana, which faces significant socio-economic hardship (The World Bank, n.d.).

Lastly, the authors emphasized that, in the context of poverty and lack of regulation, the gap between values becomes even greater, as does the weight on professional conduct. However, there is a limitation in the agency of such professionals due to the complexity of governance in the pharmaceutical industry and the intimidation exerted by institutions holding greater power, which favors capitalist morality to the detriment of health, as discussed by Vargas-Peláez et al. (2017) about how the power structures, interests, interdependencies, values and principles of the stakeholders can influence the perception towards medicines as a health necessity. To further develop health governance in Ghana, it is essential to build a collective dialogue in favor of improving governance in the pharmaceutical sector involving both governmental and private entities, instead of simply blaming the individuals included in this context.
